# Inflammatory Cytokines and Atherosclerotic Plaque Progression. Therapeutic Implications

**DOI:** 10.1007/s11883-020-00891-3

**Published:** 2020-10-06

**Authors:** Antonio V Sterpetti

**Affiliations:** 1 University of Rome Sapienza, Rome, Italy; 2 AV Sterpetti- Policlinico Umberto I, Viale del Policlinico, 00167 Rome, Italy

**Keywords:** Atherosclerosis, Inflammation, Cytokines, Growth factors, Hemodynamic forces

## Abstract

**Purpose of the Review:**

Inflammatory cytokines play a major role in atherosclerotic plaque progression. This review summarizes the rationale for personalized anti-inflammatory therapy.

**Recent Findings:**

Systemic inflammatory parameters may be used to follow the clinical outcome in primary and secondary prevention. Medical therapy, both in patients with stable cardiovascular disease, or with acute events, may be tailored taking into consideration the level and course of systemic inflammatory mediators. There is significant space for improvement in primary prevention and in the treatment of patients who have suffered from severe cardiovascular events, paying attention to not only blood pressure and cholesterol levels but also including inflammatory parameters in our clinical analysis.

**Summary:**

The potential exists to alter the course of atherosclerosis with anti-inflammatory drugs. With increased understanding of the specific mechanisms that regulate the relationship between inflammation and atherosclerosis, new, more effective and specific anti-inflammatory treatment may become available.

## Introduction

Initial research studies defined the key role played by several polypeptides, such as as platelet derived growth factor (PDGF) and basic fibroblast growth factor (bFGF), in determining smooth muscle cell growth as a response to arterial wall injury [1, 2, 3, 4•, 5, 6]. The complexity of the proliferative phenomenon was soon evident, indicating the possible involvement of other growth factors, facilitating or inhibiting the action of PDGF and b-FGF [7, 8•, 9].

The hyperplastic lesion, defined as myointimal hyperplasia, may progress in size with the accumulation of plasma-derived lipids. Several theories have been proposed to explain the accumulation of lipids in the plaque, including the action of the extracellular matrix produced by activated smooth muscle cells and the dedifferentiation of activated smooth muscle and endothelial cells in monocytes and macrophage [10, 11•]. The proliferative theory, has been challenged by the common findings of inflammatory cells in atherosclerotic plaques with a more complex structure and larger volume. T cells and other lymphocytes are present in atherosclerotic plaques. A significant role for innate (macrophages) and acquired immunity (T cells and other lymphocytes) in the progression of the atherosclerotic plaque has been hypothesized [12–15].

The atherosclerotic plaque more commonly associated with cardiovascular events has specific characteristics. Histology in autopsy studies showed, in many patients with acute coronary syndrome, a plaque with a lipid-rich necrotic tissue, with the presence of cells of innate and adaptive immunity [16–18]. These cells can be activated by inflammatory cytokines, such as interleukin (IL)1, IL2, IL 6 and TNF alfa. These inflammation mediators present multiple actions, inducing activation and chemotaxis of leucocytes, proliferation, activation, differentiation of smooth muscle cells and macrophages. Conceptually, inflammatory cytokines may facilitate the transport of lipids into the plaque, either increasing permeability of the endothelial layer or promoting differentiation of smooth muscle cells of the arterial wall in macrophages, responsible for inclusion and transport of lipoproteins [19–21].

An additional potential action of the inflammatory cytokines is the activation of vascular endothelial growth factor (VEGF). VEGF has several actions, including promoting neo vessels formation. The thin and fragile neo vessels can be disrupted, under conditions of hemodynamic stress, forming an intra-plaque hemorrhage [22–24]. Inflammatory mediators facilitate apoptosis, with the formation of necrotic tissue and consequent macrophage activation. The necrotic tissue contributes to the characteristics of the heterogeneous, complex, unstable plaque [25, 26].

The enlarging plaque can suddenly occlude the lumen of the artery. It may lead to disruptions, such as distal embolization of tissue, and exposure of blood and its coagulation factors to the substances inside the plaque, which are thrombogenic in nature [27, 28] .These inflammatory phenomena can be described as a cascade of events which, once started, regulate their own progression. Several factors have the potential to trigger the inflammatory process in vitro. The inflammatory cascade proceeds slower or faster, depending on many, often uncontrollable, stimuli, explaining the extremes of sudden occlusion of an artery or of slow chronic occlusion, which includes the possibility of compensatory mechanisms.

## Fibrous Cap Rupture

Inflammation can determine the structure of the fibrous cap, which overlies the atherosclerotic plaque [29, 30]. Rupture of the fibrous cap depends on the imbalance between the stress determined by the volume of the plaque and the intrinsic structural resistance of the fibrous cap to such a stress. A sudden increase in the internal stress related to an acutely enlarging plaque either from an intraplaque hemorrhage or by rapid accumulation of necrotic tissue can lead to the rupture of the fibrous cap. The structure of the fibrous cap is time dependent; long-term, steady and slow progression of the atherosclerotic plaque allows the development of a compact multi layered fibrous cap, with conceptually higher resistance. A sudden increase in plaque dimensions, results in a ‘weaker’ fibrous cap. Inflammatory mediators determine the production of several proteases by smooth muscle cells and by monocytes and macrophages. MMP1, MMP9 production and release is associated with inflammatory markers. At the same time, inflammation mediators inhibit TIMPs production, which neutralize proteases action [27, 31, 32].

The final event is digestion of the collagen-composed extracellular matrix, with reduced resistance to pressure by the fibrous cap.

## Sources of Inflammatory Cytokines

**Fig. 1:**
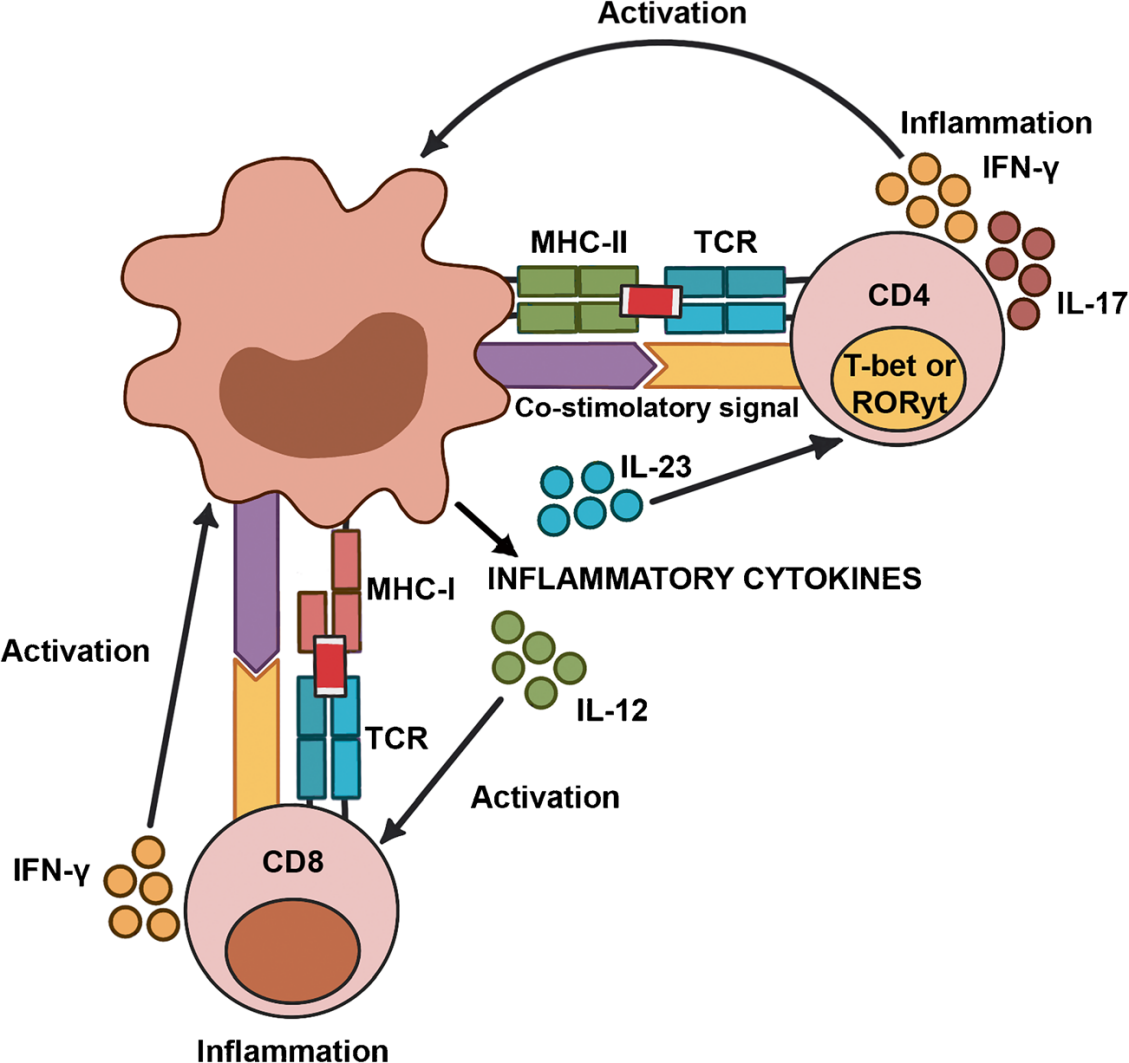
Cells of the atherosclerotic plaque (endothelial cells, smooth muscle cells, macrophages) respond to inflammatory mediators. At the same time, all cells of the atherosclerotic plaque can produce inflammatory mediators

The source of the inflammatory cytokines is generally related to the local pro-inflammatory stimuli. Many gene mutations associated with atherosclerosis evoke inflammatory responses [33, 34]. Inflammation is a physiological defence response to contrast insults. When the stimuli for inflammation persist, or the reparative action is out of control, a condition of chronic inflammation may occur. Chronic inflammation is a well-recognized risk factor for atherosclerosis development and progression [35•, 36]. The atherosclerotic plaque itself can promote and facilitate the action and continuation of the initial inflammatory stimuli. Cells in the atherosclerotic plaque (endothelial cells, smooth muscle cells, activated macrophages) can produce chemotactic factors that recruit macrophages and neutrophils (Fig. 1). Atherosclerotic cells produce high amounts of lactic acid, which is responsible for the acidification of the microenvironment [37•, 38]. The increased metabolism related to their accelerated proliferation requires significant oxygen consumption. The resulting acidification and hypoxia induce the production of inflammatory cytokines and angiogenic growth factors, which give rise to neo-angiogenesis and lymph-angiogenesis, supporting the persistence of chronic inflammation [39]. As critical examples, when compared with control animals, TNF-α and Apo E double-knockout mice have less atherosclerosis and reduced endothelial adhesion [40, 41]; an effect replicated in ApoE mice treated with agents that reduce TNF-α activity [42]. Other studies indicate that lack of IL-1β decreases the severity of atherosclerosis in ApoE-deficient mice, and antibodies targeting mouse IL-1 have resulted in reduced atherogenesis; whereas, exogenous IL-1β increases intimal medial thickening [43–45] .

### 1 Autoimmune Hypotheses

A subgroup of patients with larger plaques have an increased population of CD4^+^ T cells. The expression of class II antigens on neighbouring cells provided evidence for the functional activity of these lymphocytes in the arterial plaque [46, 47]. Several hypotheses have been presented, including the possibility that the lipids inside the plaque could represent an autoimmune stimulus. The results of the cardiovascular inflammation reduction trial (CIRT) showed that a low dose of methotrexate did not reduce circulatory levels of several pro-atherogenesis cytokines and there was not a reduction of cardiovascular events in the treated group [48]. Inflammatory and immune responses can operate to promote disease, or to promote resolution or repair of lesions. B1 lymphocytes exert an anti atherogenesis effect, but B2 lymphocytes tend to aggravate atherogenesis. Interleukins have different actions on inflammation and atherosclerosis progression; IL10 and IL16 seem to have a protective effect [49, 50].

### 2 Distant Sources

Epidemiological studies have found an association between several conditions characterized by local or systemic infections and inflammation (chronic pulmonary infections, dental infections, urinary tract infections, herpes virus infections) and increased systemic levels of inflammatory cytokines associated with atherosclerosis [51–55]. This finding supports the possibility that ‘distant’ stimuli might have a local effect on aged-related disorders, an effect defined by Libby et al.’s ‘echo effect’ [56••]. A systemic effect is also possible. In patients with human immunodeficiency virus (HIV), a link between chronic infection, immune deregulation and age-related diseases, including cardiovascular events, is possible. Microbial communities in the mouth have been shown to cause infectious diseases such as gingivitis and to be associated with increased prevalence of systemic disorders such as atherosclerosis and cancer. Cytomegalovirus infection is related to high levels of circulating inflammatory cytokines and to increased prevalence of classic age-related diseases, including atherosclerosis [57, 58].

### 3 Central Systemic Sources

Inflammatory cytokines can exert their action locally, stimulating cell growth, apoptosis and reduced removal of necrotic tissue; conceptually, it is possible that elevated levels of inflammatory cytokines might lead to a de-regulation of the immune system through a systemic effect. Both the innate and the adaptive arm of the immune system undergo marked changes with age, a phenomenon to which has been given the unspecific definition as ‘the process of immune-senescence’. With ageing, functioning of the adaptive immune system generally declines. Immuno-senescence is characterized by a thymic involution with lower production of naïve T-cells, and less sophisticated T-cell repertoire [59].

**Fig. 2:**
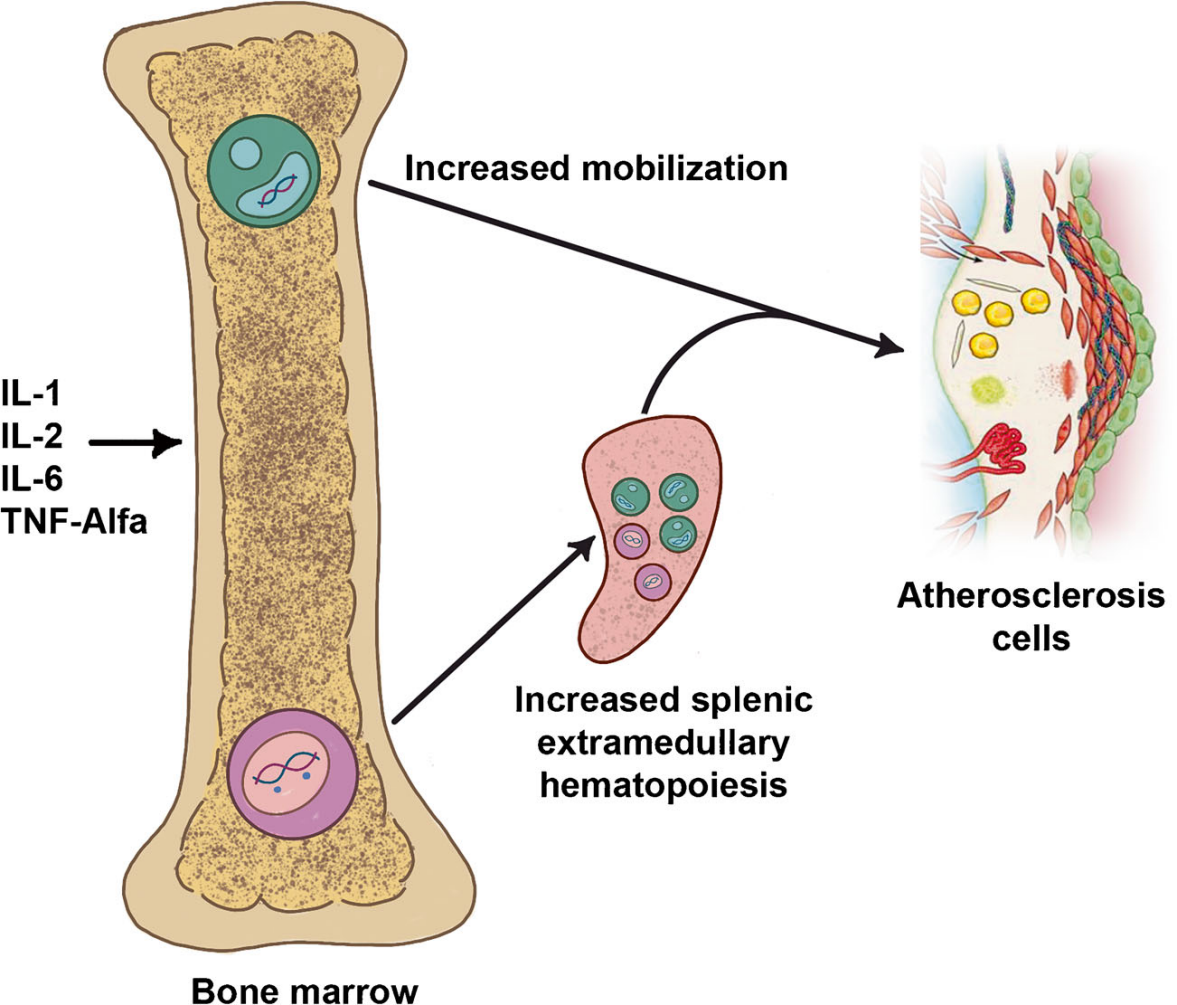
Hypotheses about how long-standing systemic inflammatory mediators might stimulate with aging the abnormal proliferation of selected medullary clones, able to favour the progression of the atherosclerotic plaque, either through the production of growth factors, or through a deviated autoimmune action. Still, there is no scientific evidence that clonal hematopoiesis occurs because of cytokine stimulation, or that bone marrow and extra medullary hematopoiesis in the spleen might involve the somatic mutations that cause clonal hematopoiesis

Despite this logical deterioration of the immune system, the innate immune system seems hyper-active in association with several degenerative diseases, as evidenced by increased serum levels of inflammatory cytokines such as IL-6, TNF-α and acute phase proteins, in patients with diffuse atherosclerosis [60•, 61]. It is possible that systemic chronic inflammation deregulates the central immune system, with the activation of an abnormal immunological response (Fig. 2). Chronic inflammation may represent a general stimulus for a deranged hematopoietic stem cell, with the possibility of proliferation of selected clones of genetically modified cells. Many of the elderly patients will host hematopoietic cell clones for prolonged periods of times without developing any clinical problem [62••, 63, 64]. This condition has been named clonal hematopoiesis of indeterminate potential (CHIP). An initial observation, however, highlighted a significant increased risk from cardiovascular events in patients who carried CHIP involving approximately 20% of their circulating blood cells.

### 4 The Importance of Hemodynamic Forces and Turbulence

**Fig. 3:**
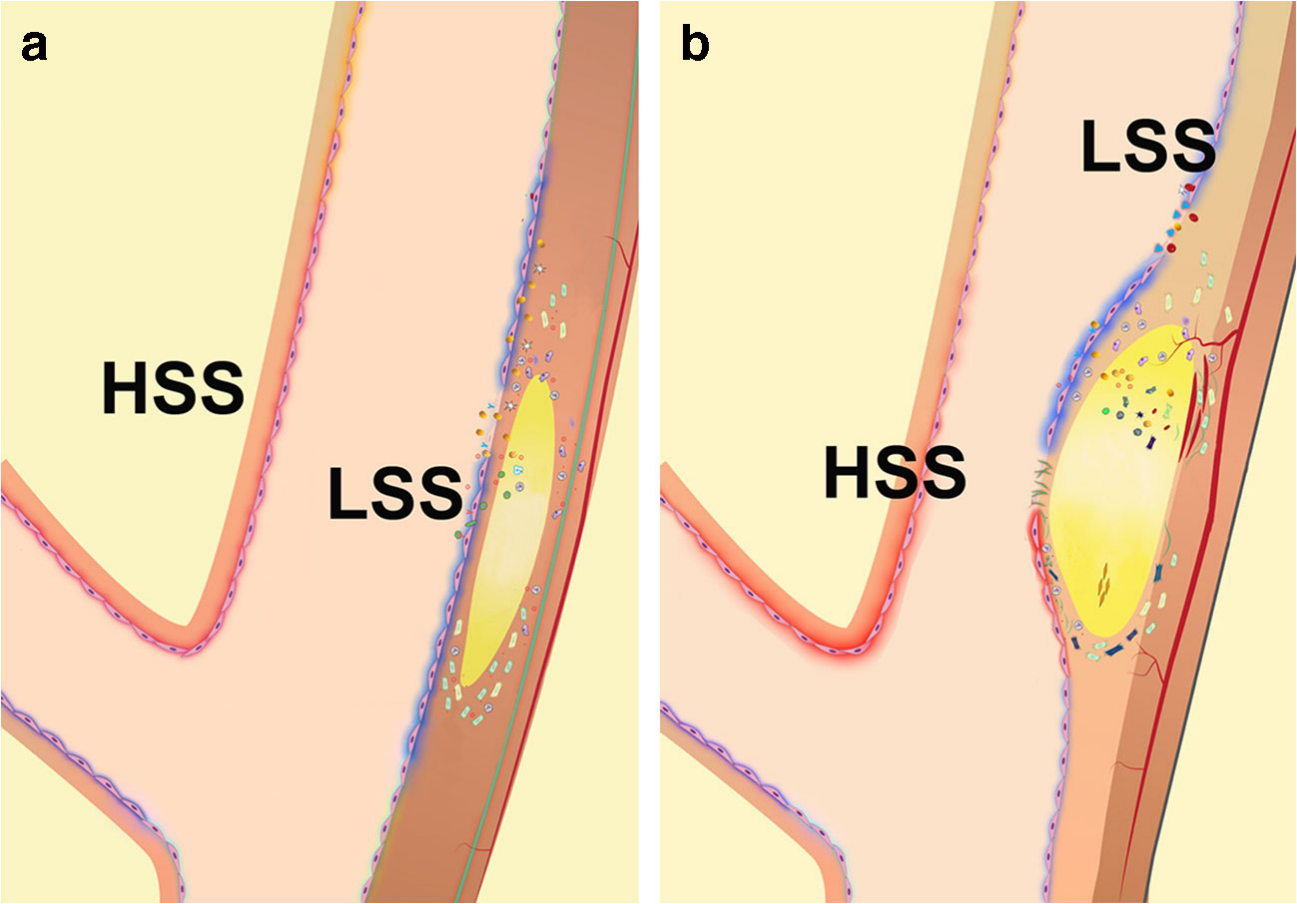
**a**, **b** Abnormal flow dynamics at the level of the bulb of the carotid bifurcation. The simultaneous presence of high shear stress (HSS) and low shear stress (LSS) determine a favourable condition for the proliferation of endothelial and smooth muscle cells, as well as for the incorporation of low density lipoproteins in the arterial wall. In areas subjected to high shear stress, there is increased production of growth factors and inflammatory cytokines which act on the neighbour regions subjected to low shear stress (modified from Thondapu et al [69]

The topographic localization of the atherosclerotic plaque suggests a potential role for hemodynamic forces in the development and distribution of atherosclerosis [65, 66]. Flow separation, with the simultaneous occurrence of areas of high and low shear stress, has been shown to be present at the level of areas predisposed to develop the atherosclerotic plaque, such as the origin or the bifurcation of the carotid and coronary arteries, or arterial segments with a sharp curvature [67]. Several studies have correlated abnormal flow dynamics to increased production of growth factors, which could explain the initial abnormal proliferation of smooth muscle cells and endothelial cells, leading to myointimal hyperplasia [68, 69] (Fig. 3).

### 4 a Collagen and Elastin Degradation

The atherosclerotic plaque occurs preferentially in regions of disturbed flow, with significant stress to the wall. Under long-term pulsatile radial pressure, elastin and collagen can lose their structural integrity. Deterioration of collagen and elastin per se activate a significant inflammatory reaction [70].

### 4 b Simultaneous Presence of High and Low Levels of Shear Stress and Production of Inflammatory Cytokines

High levels of shear stress induce the release of inflammatory mediators from endothelial cells [71, 72]. PDGF, b-FGF and VEGF production is also increased in areas of high shear stress and high radial pressure. Several in vitro and in vivo studies have shown that high shear stress has a protective effect on atherosclerosis formation [73, 74]. In conditions of high shear stress, despite the increased presence of mitogens such as PDGF, bFGF and VEGF, endothelial and smooth muscle cells tend to have a limited proliferation rate. The atherosclerotic plaques form and progress in areas of low shear stress. However, atherosclerosis does not form in the venous system, where the blood flows at low velocity with consequent low shear stress. In the venous flow, the concentration of growth factors is much reduced. In vitro studies show that endothelial and smooth muscle cells, not exposed to any shear stress, have a much higher proliferation rate in the presence of the same concentrations of growth factors, compared with cells subjected to shear stress [75, 76]. Thus, the simultaneous presence of low and high shear stress, as present in turbulence, facilitates the action of growth factors; growth factors production is increased in areas of high shear stress, and they can act, through a paracrine mechanism, in the neighbour cells, located in the low shear stress zone, where they can easily proliferate.

Endothelial and smooth muscle cells situated in areas of low shear stress, in the context of a turbulence zone, can easily proliferate. A similar action mechanism is associated with the increased production of IL-1, IL-2, IL-6 and TNF alfa in areas of high shear stress; these cytokines may exert their inflammatory action on the neighbour cells, subjected to a low shear stress. The inflammatory action may promote proliferation of the endothelial and smooth muscle cell, and may facilitate LDL transport, through increased permeability of the endothelial cells and differentiation of smooth muscle cells in monocytes and macrophages involved in LDL transport. The involvement of the inflammasome complex may lead to oxidation of LDL. The transport of LDL in a zone of low shear stress is facilitated by the accumulation in this zone of LDL, in a general condition of turbulence [69, 77–80].

### Ultrasound studies

Ultrasound studies demonstrated the changes of a plaque from a homogenous structure to a complex heterogeneous structure, and vice-versa [81, 82]. In completely occluded arteries, the plaque always appeared homogenous during ultrasound. Pathology showed that the ‘heterogeneous’ structure was often correlated to the presence of intraplaque hemorrhage and/or a large soft lipidic core [82–84]. These observations testify to the conceptual possibility that the inflammatory reaction precedes the progression from stable to unstable plaque. On the other hand, with time, an initial ‘heterogenous’, vulnerable plaque, related often to intraplaque hemorrhage, can transform into a homogenous, stable plaque, if for several reasons (mainly slow progression) a valid balance is established between the internal pressure of the plaque and a thick fibrous cap [85•].

### 4c: Direct trauma by high shear stress to the atherosclerotic plaque

In vivo and in vitro experiments have shown that high shear stress per se prevents occlusive changes in the artery. The atherosclerotic plaque forms preferentially in areas of low shear stress in the context of disturbed flow. However, once the atherosclerotic plaque has formed, high levels of shear stress, favoured by the increased flow velocity in the narrowed lumen, can directly determine a trauma to the endothelium with desquamation and exposure of the content of the plaque to the blood and its coagulation factors [22, 86]. In the case of recently developed plaque, there is the possibility that the content of the plaque is composed mainly of hemorrhage and a lipid rich core, which, for their intrinsic thrombogenic nature, can determine platelet adhesion and production of inflammatory cytokines [22, 87•, 88]. In this condition, the production of inflammatory cytokines represents a secondary event and not a causal factor. The inflammatory cascade contributes to the severity of the local and systemic clinical picture. Alternatively, if the content of the plaque is formed mainly by fibrosis and collagen, as it is commonly found in slowly formed plaques, the desquamation of the endothelium determines exposure to the blood and its coagulation factors of a material less thrombogenic; in this condition, there is mainly aggregation of platelets, forming a white thrombus with minimal inflammatory reaction. Ultrastructural studies showed increased content of proteoglycan and glycosaminoglycans, hyaluronic acid and hyaluronan receptor CD44 in chronic lesions complicated by superficial erosion [31, 32, 89–91]. Eroded chronic plaques have few inflammatory cells and many smooth muscle cells, whereas vulnerable plaques (presumably formed in a shorter time), have many mononuclear cells (macrophages and monocytes) and few smooth muscle cells. The fibrous cap shows various characteristics: in the eroded chronic plaque, the fibrous cap is thick, whereas in the unstable, acutely formed plaque, the fibrous plaque is thin. The type of thrombus associated with superficial erosion differs from the thrombus formed in the case of an unstable plaque rupture. Thrombus aspirated by percutaneous coronary techniques shows different patterns. Thrombi complicating eroded chronic lesions have a white color and they are rich in platelets, whereas thrombi complicating ruptured unstable plaques are predominantly red and rich in fibrin. The thrombi overlying ruptured unstable plaques are much richer in myeloperoxidase-positive inflammatory cells [31, 32, 89, 90, 91, 92•]. These findings suggest marked differences in the pathophysiology of erosion of chronic stable plaques from rupture of unstable plaques with thin fibrous cap. The clinical picture will depend on the characteristics of the plaque itself. In the PROSPECT (Providing Regional Observations to Study Predictors of Events in the Coronary Tree) study, intravascular ultrasound techniques were used to evaluate the character of tissue in coronary atherosclerotic plaques [84]. Serial intravascular imaging studies suggested that the morphology of human coronary atheromata evolves in time, from an unstable to a stable morphology, and vice-versa.

## Inflammatory Cytokines Production. A Primary, Causal Event, or a Secondary Event? Need for Specific Treatments

From the previous descriptions, it appears that inflammatory cytokines may have both a causal, primary effect, and a secondary, aggravating effect. Inflammatory cytokines, independently from the specific factor triggering their production, have the potential to facilitate plaque development and progression from stable, homogenous plaque to heterogenous, unstable plaque.

The rupture of heterogeneous, unstable plaque is associated with thrombosis and production of inflammatory cytokines which can aggravate the clinical picture. The limited inflammatory reaction which accompanies the white thrombus formation in the case of chronic plaque erosion explains a less severe associated clinical picture with better clinical outcome.

### Reduced Levels of Inflammatory Cytokines after Therapy

Reduced systemic levels of inflammatory cytokines after therapy is a valid prognostic factor for better clinical outcomes in patients with previous cardiovascular events. Persistence of elevated levels of inflammatory cytokines determines a poorer clinical outcome in patients who had acute coronary syndrome, despite standard optimal therapy. This evidence might be related to the correlation between persistent elevated levels of inflammatory cytokines and more aggressive forms of atherosclerosis [22].

There is also the possibility that the activation of genetically determined hematopoietic clones, by prolonged chronic inflammation, has a cut-off limit, which once overcome leaves no possibility for regression. This hypothesis brings into clinical consideration the positive effects in preventing age-related disease, such as atherosclerosis, with an aggressive approach in treating any form of acute inflammation, before the development of a condition of chronic inflammation.

### Anti-Inflammatory Therapy for Atherosclerosis

A generic, low cost anti-inflammatory and cholesterol reducing therapy with statins may play a major role in primary prevention in the elderly general population, with few side-effects [93•]. They have become a standard therapy for patients at risk for cardiovascular events; statins have the potential for an adjunctive anti-atherosclerosis effect in view of their unexpected action regarding reducing inflammation. In the JUPITER (Justification for the Use of Statins for Prevention: Intervention Trial Evaluating Rosuvastatin) study [94], patients treated with statins had reduced levels of cholesterol and reduced systemic inflammatory parameters. Clinical outcome was much better in the subgroups of patients in whom both cholesterol and inflammatory parameters were significantly reduced.

There has been a diffuse use of inhibitors of platelet activation, on the theoretical basis that platelet inhibition may prevent the thrombosis associated with plaque development and complications. Considering the detrimental consequences of intraplaque hemorrhage, in the progression in size and stability of the plaque, one could question if platelet inhibition might worsen the consequences of rupture of thin intraplaque neo-vessels.

The role of aspirin (100 mg/ day) in the primary prevention of individuals at moderate risk has been questioned, reviewing the results of three recently published trials. Two trials did not show any long-term benefit in the general elderly population at low risk for cardiovascular events who received aspirin therapy (100/mg/day) compared with the group of people taking placebo [95•, 96••]. This finding was confirmed in the ASCEND study, which included only patients with diabetes [97••, 98••, 99••]. Aspirin therapy did not have positive effects in diabetic patients at moderate risk for cardiovascular events. Aspirin had a 5-year marginal preventive effect in patients at higher risk for cardiovascular events. However, the bleeding complications were more frequent than previously reported. A recent meta-analysis underlined the possible importance of the aspirin dosage: in patients weighting more than 75 kg, the 100 mg dose was not effective, whereas it was effective in patients weighting less than 75 kg.

The possibility that a more effective anti-platelet therapy could reduce cardiovascular events in patients with stable cardiovascular disease has been tested by adding clopidogrel to aspirin (100 mg/die). Studies have shown that the association of clopidogrel reduced the number of cardiovascular events, but not the overall mortality [100, 101]. Ticagrelor, with an action similar to clopidogrel, inhibiting the receptor P2Y-12, added to a 100 mg/die dose of aspirin, resulted in a lower rate of cardiovascular events, in patients with previous myocardial infarction, without reducing the overall mortality [102]. However, there was no difference between ticagrelor and clopidogrel in patients with stable peripheral arterial disease [103•].

The negative clinical consequences of the superimposed thrombosis on the ruptured atherosclerotic plaque represent the theoretical basis to add antithrombotic therapy to antiplatelet therapy. Warfarin therapy in stable cardiovascular disease did not result in better results in patients with peripheral arterial disease, even if the overall number of cardiovascular events was reduced, at a cost of increasing bleeding rates [104, 105].

Adding vorapaxar, a thrombin-receptor agonist, to antiplatelet therapy reduced the number of major cardiovascular events in patients with stable cardiovascular disease, but not the overall mortality. In the COMPASS (Cardiovascular Outcomes for People Using Anticoagulation Strategies) trial, patients who received Rivaroxaban (2.5 mg/die), a selective factor Xa inhibitor associated with aspirin (100 mg/die), had fewer cardiovascular events (24%), compared with patients who received aspirin alone (100 mg/die) or Rivaroxaban alone (5 mg/die); bleeding rates, however, were higher [106, 107•].

A selective inhibition of the inflammatory cascade is conceptually more appropriate, considering the many and contrasting actions of the involved mechanisms. In the CANTOS (Canakinumab Anti-Inflammatory Thrombosis Outcomes Study) trial [108], patients with previous myocardial infarction and elevated inflammatory parameters were treated with an antibody able to neutralize the action of IL-1 beta. Patients treated with canakinumab had a lower incidence of new cardiac events. An equally important observation in the CANTOS study was that the magnitude of cytokine reduction achieved by individual trial participants was a major determinant of clinical efficacy. Moreover, the patients who had a more significant reduction of systemic inflammatory parameters had 31% reductions in cardiovascular and all-cause mortality (both *P* <0.001).

Several other studies have shown improved results after open and endovascular surgery, when patients had double and even triple antiplatelet therapy [109, 110, 111•]. In general, these improved results have been attributed to the action on platelets. Is it not possible that the positive effects of this aggressive antiplatelet therapy depend on their associated anti-inflammatory action? Although our present knowledge is rudimentary, the potential exists to alter the course of atherosclerosis with anti-inflammatory drugs. With increased understanding of the specific mechanisms that regulate the relationship between inflammation and atherosclerosis, new, more effective and specific anti-inflammatory treatment may become available.

Conceptually, more valid drugs should have a multiple and specific anti-inflammatory action. The inflammatory cascade is complex and implies many regulatory mechanisms. A selective inhibition could have only a temporary effect, being easily overcome by compensatory, alternative routes in the context of a chronic, long-standing process. Several pharmacological agents are available that alter IL-1 function, including anakinra (an IL-1 receptor antagonist that inhibits IL-1α and IL-1β) and rilonocept (an IL-1 trap that further inhibits the IL-1 receptor). The MRC-ILA (Medical Research Council Interleukin-1 Antagonist) heart study (a biomarker and not a clinical evaluation) [112] showed initial promise for 2-week anakinra treatment in non-ST-segment elevation acute coronary syndromes but with little long-term benefit.

## The Neglected Importance of the Residual Inflammatory Risk

Controlled randomized studies, performed in academic centres, have shown a significant number of patients with high levels of systemic inflammatory parameters despite standard optimal therapy. In the PROVE-IT (Pravastatin or Atorvastatin Evaluation and Infection Therapy) trial [113] and in the IMPROVE-IT (Improved Reduction of Outcomes: Vytorin Efficacy International) trial [114], in approximately 40% of the patients who had atorvastatin therapy, both the cholesterol levels and the inflammatory parameters were significantly reduced. In these trials, the number of patients with persistent high inflammatory parameters (defined as on treatment hsCRP>2 mg/L) was much more common than the number of patients with persistent high levels of LDL (defined as an on-treatment LDLC<70 mg/dL). Controlled trials in academic centres involve a careful follow up of the patients, who inevitably are very motivated to adhere to the prescribed therapy. National data from the VIRGO (Variation in Recovery: Role of Gender on Outcomes of Young AMI Patients) registry [115•] show that 60% of the analysed patients had residual inflammatory risk. There is significant space for improvement in the treatment of patients who suffered from severe cardiovascular events, paying attention to not only blood pressure and cholesterol levels but also including inflammatory parameters in our clinical analysis.

## Conclusions

A careful control of blood pressure, cholesterol levels, sugar levels and resorting to medications only when strictly needed, should be considered the optimal standard therapy. An aggressive, general control and treatment of any form of inflammation and infection may lead to reduced cardiovascular events in the general population. In patients who have had a cardiovascular event, or present major risk factors, a careful analysis and correction of cholesterol and inflammatory parameters should be considered.

Epidemiological studies have shown that blood pressure control, and consequent lower dynamic stress to the arterial wall, reduces the prevalence of cardiovascular events.

Considering the several physiopathological mechanisms which characterize the different stages of the atherosclerotic plaque development and progression, as well as the multiple actions correlated to cardiovascular events, the principle that each patient should be considered in his/her individuality in choosing the most appropriate treatment remains a cornerstone for an optimal treatment and better clinical outcomes.

In this context, medical therapy, both for primary or secondary prevention, as well as in the acute phase of the cardiovascular event, should be chosen also taking into consideration the inflammatory parameters.

Another significant point to be made is the underestimated severity of the complications related to antiaggregant and anticoagulation therapy. The differences between academic trials and the real medical world are well known. In academic trials, patients are carefully followed and inevitably selected according to many criteria, including their mental status, capability to be self-sufficient in daily activities and to be motivated.

The daily experience in the emergency room, and from national data of the general population, show that the number of patients taking antiplatelets admitted or seen for gastric bleeding is steadily increasing, namely in the elderly population [116].

At the same time, national statistics show an increasing mortality for head trauma, especially resulting from falls involving elderly patients [117, 118•]. A cerebral hemorrhage in the elderly, taking antiplatelet therapy, and not adequately followed by the family members or caretakers, can easily progress to coma, before attention is paid to the clinical conditions. These increased mortalities and complications in the elderly may be related to the widespread, often uncontrolled, use of antiaggregant therapy.

## Future Perspectives

The negative aspects of chronic inflammation, as a risk factor either for progression of atherosclerosis or for cancer occurrence, are supported by a series of new basic and clinical studies. In the elderly, aspirin has been shown to determine often uncontrollable side effects, mainly related to its inhibitory action on platelets. The possibility also exists that the anti-platelet action of aspirin may favour the progression of the atherosclerotic plaque from a homogenous to a heterogeneous pattern predisposing to intraplaque hemorrhage. Considering the double action of statins, either as lipid-lowering or anti-inflammatory agents, the role of aspirin in the prevention of cardiovascular events should be reconsidered in elderly patients taking statins. While platelet inhibition may play a major role in secondary prevention, its role in primary prevention may not be so evident, except in specific clinical settings.

New, more selective anti-inflammatory agents may play a major role either in primary or secondary prevention opening an important field for research.
